# The Damage of the Crayfish (*Procambarus Clarkii*) Digestive Organs Caused by *Citrobacter Freundii* Is Associated With the Disturbance of Intestinal Microbiota and Disruption of Intestinal-Liver Axis Homeostasis

**DOI:** 10.3389/fcimb.2022.940576

**Published:** 2022-07-05

**Authors:** Minghao Li, Jincheng Wang, Huiling Deng, Liangyu Li, Xiaoli Huang, Defang Chen, Ping Ouyang, Yi Geng, Shiyong Yang, Lizi Yin, Wei Luo, Jun Jiang

**Affiliations:** ^1^ Department of Aquaculture, College of Animal Science & Technology, Sichuan Agricultural University, Chengdu, China; ^2^ Fishery Research Institute, Chengdu Academy of Agriculture and Forestry Sciences, Wenjiang, Sichuan, China; ^3^ Department of Basic Veterinary, College of Veterinary Medicine, Sichuan Agricultural University, Chengdu, China

**Keywords:** *Citrobacter freundii*, crayfish, intestinal-liver axis, intestinal microbiota, RNA-Seq

## Abstract

As a common conditional pathogenic bacterium in nature, *C. freundii* has posed a threat to crayfish culture and may infect humans through consumption. However, the pathogenic mechanism of *C. freundii* in crayfish remains unknown, which poses difficulties for the prevention and control of the bacterium. In this study, the effects of *C. freundii* on the digestive organs, intestine and hepatopancreas, of crayfish were investigated by high-throughput sequencing technology combined with histological analysis and flow cytometry. The findings suggested that *C. freundii* caused disruption of the intestinal microbiota, leading to intestinal inflammation and disrupting intestinal integrity. Meanwhile, *C. freundii* infection stimulates bile acid biosynthesis in the intestinal microbiota. Transcriptomic results showed significant upregulation of hepatopancreatic lipid degradation pathway and cytochrome P450-related pathways. Follow-up experiments confirmed a decrease in intracellular lipids and an increase in ROS and apoptosis. All the results indicated the disruption of intestinal-liver axis homeostasis due to disturbed intestinal microbiota may as a potential basis for *C. freundii* pathopoiesis in crayfish. These results provide new insights into the pathogenic molecular mechanisms of *C. freundii* in the infection of crayfish.

## 1 Introduction

The red swamp crayfish (*Procambarus clarkii*) is a species of freshwater economic crayfish that is native to North America and widely distributed in nature. Crayfish are delicious, nutritious, adaptable and fast breeding. After its introduction to China, it quickly became one of the most important commercial aquaculture species, with production reaching 2.4 million tons in 2020 (FAO). However, diseases caused by viruses and bacteria, such as *Vibrio Parahemolyticus* and white spot syndrome virus, have caused huge losses to the crayfish farming industry (Dong et al., 2016; Pace et al., 2016).

*Citrobacter freundii* (a bacterium normally found in the intestinal tract) is a conditionally pathogenic bacterium that has recently been reported to infect crayfish and cause mortality (Liu et al., 2020). It belongs to the genus *Citrobacter* in the family Enterobacteriaceae and is widely distributed in nature. Previous reports have shown that *C. freundii* is a pathogenic bacterium that poses a serious threat to aquaculture and is highly pathogenic to economically farmed species such as rainbow trout, eel, and Chinese sturgeon (Aydin et al., 1997; Joh et al., 2013; Yang et al., 2021). More worryingly, in addition to aquatic animals, *C. freundii* is also sensitive to mammals and can cause meningitis, endocarditis and other diseases to neonates and horses (Guidi et al., 2016; Chen and Ji, 2019). Moreover, there is also a risk of food poisoning when people consume aquatic products infected with *C. freundii* (Pletz et al., 2018). However, although *C. freundii* has posed a threat to the crayfish culture industry, the pathogenic mechanism of *C. freundii* on crayfish still remains relatively unknown, which poses a great obstacle to the prevention and control of the bacterium.

The intestine-liver axis refers to the two-way relationship between the intestine, also its microorganisms and the liver, which is established through the portal vein. The intestinal-liver axis in health is involved in the nutrient absorption and immune response of the organism (Albillos et al., 2020). Bile acids are an important metabolite linking the intestine and liver, and are secreted from the liver to the intestine to facilitate the digestion of nutrients. Most (~95%) of the bile acids are reabsorbed into the hepatic portal vein of the liver, while the unabsorbed bile acids are converted to secondary bile acids as substrates for microbial metabolism (de Aguiar Vallim et al., 2013). However, intestinal microbiota dysbiosis can lead to intestinal inflammation and increased intestinal permeability, resulting in increased exposure of the liver to intestinal bacteria and their products (Chen et al., 2021). Growing evidence indicates the pathogenetic role of microbe-derived metabolites, such as secondary bile acids in the pathogenesis of liver disease (Ferslew et al., 2015). Notably, some researchers have shown that dysbiosis of the intestinal microbiota is closely associated with pathogenic microbial infections (Li et al., 2020). Some studies reported the intestine-liver axis in humans and mice, but this has remained largely unexplored in aquatic organisms, especially in the case of pathogenic infections.

In the present study, we used high-throughput RNA-sequencing (RNA-seq) and high-throughput 16S rRNA sequencing to detect the response of hepatopancreas and changes of intestinal microflora in crayfish infected with *C. freundii*. Combined with the sequencing results and histological changes, we hope that the findings will help us to better understand the pathogenic mechanism of *C. freundii* on crayfish and provide a theoretical basis for the control of the bacterium.

## 2 Materials and Methods

### 2.1 Crayfish and Bacteria

Crayfish (average weight 16.18 ± 1.10 g, random sex) were purchased from the local market in Sichuan Province (China). Prior to the experiment, the crawfish were kept individually in a 19 cm × 12.5 cm × 7.5 cm plastic box for one week to acclimate to the experimental conditions. During the acclimatization, the rearing temperature was kept at 26°C and photoperiod was maintained at 14 hours of light and 10 hours of dark. The crayfish were fed twice a day with commercial diets and dried mealworms (*Tenebrio molitor*) and the water was exchanged one third of the total once every day with fully aerated tap water. 120 healthy crayfish with good vitality were randomly divided into control group and infection group.

The pathogen *C. freundii* strain was isolated and purified from hepatopancreas in moribund red swamp crayfish. Next, the strain was identified as *C. freundii* by 16S rDNA sequencing. Isolates were preserved in 50% (v/v) glycerol at −80°C. After resuscitating the purified bacterial strain, the strain was inoculated into an LB Broth Medium with constant shaking at 28°C for 24h. The broth culture was centrifuged at 8000 rpm for 10 min at 4°C to collect the sediment. The bacterial pellet was washed with sterile phosphate buffered saline (PBS) and re-suspended in PBS at a final concentration of 2.7×10^5^ CFU/mL (one-tenth of the LD_50_), in preparation for the experimental infection (Feng et al., 2021).

### 2.2 Experimental Infection

In the experimental infection test, 100μL samples of the 1/10 LD_50_ dilutions were injected into crayfish of infection group near the third abdominal segment. The crayfish in the control group were injected with an equal volume of sterile PBS. During the 96-hour experiment, fresh moribund crayfish from the infected group were collected for bacterial isolation and the isolated bacteria were identified by 16S rDNA sequencing to confirm successful infection. The experimental conditions during experimental infection were consistent with the period of acclimatization.

### 2.3 Samples Collection

In the process of the infecting trial, the crayfish were sampled at 96h post-injection. The appearance and organ of crayfish were photographed and recorded. The hepatopancreas and intestine of crayfish were fixed with Davidson’s AFA fixative for histological observation. Hepatopancreatic transcriptome samples and intestinal microbial samples were frozen in liquid nitrogen and stored at -80°C until RNA or DNA extraction was performed. To reduce errors caused by sampling, all frozen samples were guaranteed in at least three biological replicates and each biological replicate was composed of a mix of tissues from three to five individuals.

### 2.4 Histopathological Analysis

The hepatopancreas and intestine of crayfish from the two groups were fixed with Davidson’s AFA fixative for at least 48 h for histological observation. The samples of all tissues were trimmed into cassettes, dehydrated in graded ethanol solutions, cleared in xylene, embedded in paraffin wax, sectioned at 4 µm, mounted and dried on slides and stained with hematoxylin and eosin (H&E). Histopathological changes were observed under an optical microscope (Nikon, Tokyo, Japan) after staining.

The degree of hyperplasia, necrosis, inflammatory cell infiltration, vacuolization, and deformation of different organs were graded according to the scoring system proposed by Baums and colleagues (Baums et al., 2013). Histological changes were assessed using a score (S) ranging from 0 to 6, depending on the extent and extent of the lesion: (0) unchanged; (2) mild occurrence; (4) moderate occurrence; and (6) severe occurrence (diffuse lesion).

### 2.5 Oil Red O Staining

Small pieces of hepatopancreas (0.5 × 0.5 × 0.5 cm^3^) were fixed in liquid nitrogen and stored frozen at -80°C. After embedding into the optimal temperature compound, the embedding agent was placed in a cryostat for section (5 μm). Neutral lipid staining was performed using the Oil Red O staining kit (Sigma-Aldrich, Beijing, China) according to the manufacturer’s instructions. Measurement of lipid staining area after observation under optical microscope using ImageJ software.

### 2.6 Flow Cytometry Assay

Three crayfish were randomly selected from each group, and the hepatopancreas was dissected and placed in iced PBS (0°C). The hepatopancreas was immediately minced to form a cell suspension and filtered through a 300-mesh nylon screen. Cells were washed twice with cold PBS, and the cell pellet was resuspended in PBS. The cells were detected using Cyto FLEX flow cytometry. The ROS was detected using the cell suspension and cell apoptosis was detected using Annexin V-FITC (Thermo, Shanghai, China).

### 2.7 RNA-Seq

#### 2.7.1 RNA Extraction and cDNA Library Construction

Total RNA was extracted from the hepatopancreas using TRIzol^®^ Reagent, and its integrity and purity were detected by 2100 Bioanalyser (Agilent Technologies, Inc., Santa Clara CA, USA). ND-2000 (NanoDrop Thermo Scientific, Wilmington) was used for quantification. Only high-quality RNA sample (OD260/280 = 1.8~2.2, OD260/230≥2.0, RIN≥8.0, 28S:18S≥1.0, >1μg) was used to construct sequencing library.

Hepatopancreas RNA-seq transcriptome libraries were prepared using Illumina TruSeqTM RNA sample preparation Kit (San Diego, CA). mRNA was enriched with magnetic beads with Oligo (dT) and fragmented with a fragmentation buffer. cDNA was synthesized using mRNA as a template. Then the synthesized cDNA was subjected to end-repair, phosphorylation and ‘A’ base addition according to Illumina’s library construction protocol. Libraries were size selected for cDNA target fragments on 2% Low Range Ultra Agarose followed by PCR amplified using Phusion DNA polymerase (New England Biolabs, Boston, MA) for 15 PCR cycles. The effective concentration of the library was quantified by TBS380, and transcriptome sequencing was conducted using Illumina Hiseq xten/NovaSeq 6000 sequencer (Illumina, San Diego, CA) according to the effective concentration.

#### 2.7.2 *De Novo* Assembly and Transcriptome Analysis

Clean reads were obtained by removing the subassembly and low-quality sequences, and the transcripts were performed with clean reads after merging with Trinity software. BOWTIE software was used to compare fragments of each sample to transcripts and the abundance information of each fragment was statistically analyzed. All the assembled transcripts were searched against the NCBI Nr, GO, Pfam, KEGG, COG and Swiss-Prot databases using BLASTX (E-values ≤1e −5) to obtain the annotation information. Fragments/KB/Million reads (FPKM) were used to analyze the expression levels of differential genes. The statistical analysis of differentially expressed genes (DEGs) was performed using the DESeq2 package with FDR<0.05 & |log2FC|≧1 as the default screening criteria. In addition, the functional enrichment analysis of the screened DEGs was performed by Goatools and KOBAS with corrected P-value ≤ 0.05 to determine the extent of DEG enrichment in different GO terms and KEGG pathways.

### 2.8 Intestinal Flora Analysis Procedure

#### 2.8.1 DNA Extraction and PCR Purification

Microbial community genomic DNA was extracted from hepatopancreas samples using a bacterial DNA isolation kit (Foregene Co., Ltd., China) according to the manufacturer’s instructions. After extraction, the genomic DNA was detected by 1% agarose gel electrophoresis. The hypervariable region V3-V4 of the bacterial 16S rRNA gene were amplified with forward primer (338F: 5’- ACTCCTACGGGAGGCAGCAG-3’) and reverse primer (806R: 5’- GGACTACHVGGGTWTCTAAT-3’). The PCR product was extracted from 2% agarose gel and purified using the AxyPrep DNA Gel Extraction Kit (Axygen Biosciences, Union City, CA, USA) according to manufacturer’s instructions and quantified using Quantus™ Fluorometer (Promega, USA).

#### 2.8.2 Sequencing and Processing

Purified amplicons were pooled in equimolar and paired-end sequenced on an Illumina MiSeq PE300 platform/NovaSeq PE250 platform (Illumina, San Diego,USA) according to the standard protocols by Majorbio Bio-Pharm Technology Co. Ltd. (Shanghai, China).

After demultiplexed, the raw 16S rRNA gene sequencing reads were quality-filtered by fastp version 0.20.0 and merged by FLASH version 1.2.7. The dataset was prepared for analysis by excluding that: (i) reads shorter than 50 bp or containing ambiguous characters, (ii) overlapping sequences shorter than 10 bp, (iii) primer mismatch number was greater than two nucleotides. Operational taxonomic units (OTUs) with 97% similarity cutoff were clustered using UPARSE version 7.1, and chimeric sequences were identified and removed. Each OTU was compared with the 16s rRNA database (Silva), using BLAST analysis, to obtain species classification information. Species with relative abundance less than 0.01 in the samples were classified as “other”.

### 2.9 Statistical Analysis

All experiments were performed three times or in triplicate and all data were expressed as mean ± standard deviation. Student’s t-test and Wilcoxon rank-sum test were conducted on the experimental data. Statistical analyses were performed using IBM SPSS 26.0 (SPSSInc, Chicago, USA), and statistical significance was defined as P<0.05.

## 3 Results

### 3.1 Infection Caused Severe Disturbance of the Intestinal Microbiota and Intestinal Inflammation

We first focused on the changes in the intestine after infection, as *C. freundii* is mainly present in the animal intestine under normal conditions. To investigate the effect of *C. freundii* on the intestinal microbiota, 16s rRNA high-throughput sequencing was performed.

**Figure 1:**
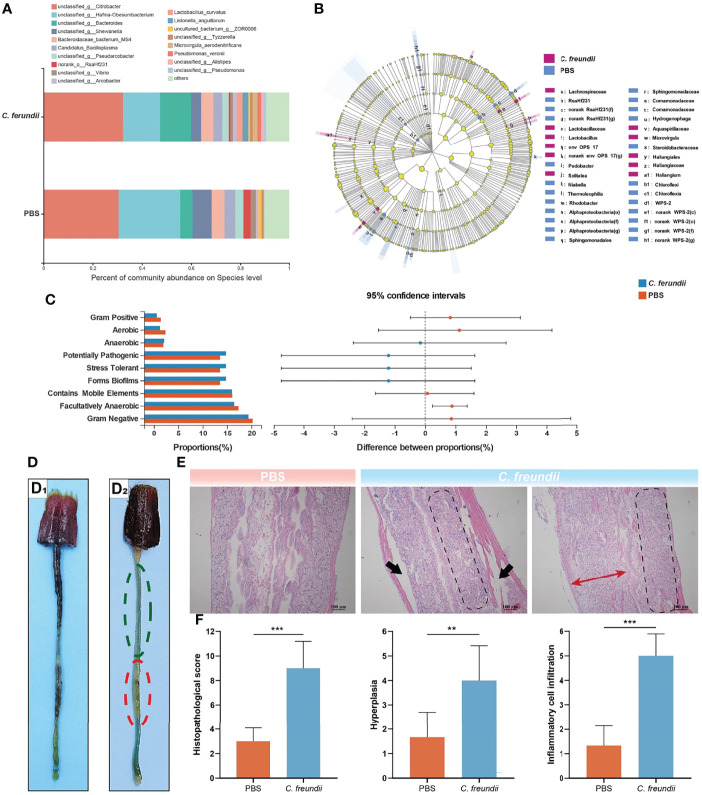
Effects of *C. freundii* infection on intestinal microbiota composition and intestinal structure. **(A)** Intestinal microbiota composition at the species level in different groups of the crayfish. **(B)** Taxonomic representation of differences in the intestinal microbiota of crayfish injected with PBS and *C. freundii*. The concentric circles from the inside out represented the different taxonomic classes (phylum to genus). The different coloured nodes indicated differences in intestinal microbiota (Red represented significantly higher abundance of intestinal microbiota in the infection group than in the control group, while blue represented the opposite, and yellow indicated no significant difference). The size of each node indicated the abundance of intestinal microbiota. **(C)** Prediction of microbial community phenotype based on BugBase. **(D)** General characteristics of the intestine of crayfish in the control (D_1_) and infected groups (D_2_). More areas of intestinal inflammation (Green dotted circle); The intestinal segment was inflated and swollen (Red dotted circle). **(E)** Histopathological observations on the intestine of the crayfish (H&E staining). The muscularis is separated from other tissue (Black arrow); Intestinal wall cell hyperplasia (Red bidirectional arrow); Inflammatory cell infiltration (marked by parallelogram shape). **(F)** Intestinal histopathological score. (*:p < 0.05; **:p < 0.01; ***:p < 0.001).

The results showed that infection altered the alpha diversity of the intestinal microbiota, although not to reach statistical significance (Table S1). At the phylum level, Proteobacteria, Bacteroidota, Firmicutes and Campilobacterota were the dominant species in both groups of samples, but their relative abundance differed between the two groups (**Figure S1A**). The relative abundance of Proteobacteria and Firmicutes in the control group was higher than that of the infected group, but the relative abundance of the Bacteroidota in the infected group samples was higher than that of the control group and the difference was the largest. At the genus level, *Hafnia-Obesumbacterium* and *Shewanella* were dominant in the control group, while Bacteroides was dominant in the infected group (**Figure S1B**). Intriguingly, we noted a higher abundance of many pathogenic bacteria in the infected group than in the control group, including Bacteroides, Vibrio, Pseudomonas and Arcobacter. The species-level annotation results were similar to the genus level, with *Citrobacter* being the dominant species in the intestine (**Figure 1A**). Linear discriminant analysis (LDA) effect size analysis (LEfSe) was used to further investigate the changes in the intestinal flora. The analysis showed that the relative abundance of more bacteria in the intestine of crayfish showed a trend of reduction after *C. freundii* infection (**Figure 1B**).

Moreover, BugBase was used to predict the phenotype of intestinal microorganisms. The phenotypes of the two groups were compared using the Wilcoxon rank sum test and corrected for Bonferroni. The results showed that the relative abundance of microorganisms with Gram-positive, Aerobic, Gram-negative, Contains Mobile Elements and Facultatively Anaerobic phenotype in the intestine of the control group were higher than that in the infected group (**Figure 1C**). In contrast, the relative abundance of the Anaerobic and Stress Tolerant phenotype was higher in the infected group. Notably, in addition to the Anaerobic and Stress Tolerant, disease-related phenotypes such as Potentially Pathogenic and Forms Biofilms also had higher relative abundance in the infected group, suggesting that *C. freundii* infection may cause more pathogenic bacteria to colonize the crayfish intestine.

The above results of 16s rRNA high-throughput sequencing indicate that *C. freundii* infection alters the intestinal microbiota of crayfish. Interestingly, those research about the pathogenesis of inflammatory bowel disease (IBD) has showed that the disturbances in the intestinal microbiota were the principal factors that caused IBD (Zhang and Li, 2014). In this study, however, compared to the control group, it also showed the infected group had more blue areas (inflammatory areas), lack of food filling in the intestine, and some of the intestinal segments were slightly inflated and swollen (**Figure 1D**). The control intestine showed typical structures, including the epithelium, lamina propria, submucosa, and muscularis, while there were no obvious signs of injury or inflammation. However, the intestine of the infected group exhibited significant damage. Specifically, the muscularis of the intestine infected with *C. freundii* was partially separated from the submucosa, and the intestinal wall was partially hyperplastic and infiltrated by a large number of inflammatory cells (**Figure 1E**). Histopathological scores showed significant damage to the intestine of crayfish caused by the infection, which mainly in the form of moderate to severe enteritis accompanied by cellular hyperplasia (**Figure 1F**). All these results indicated that the disturbance of intestinal microbiota combined with the infected *C. freundii* could lead to intestine injury, causing moderate to severe necrotic enteritis.

### 3.2 Infection Changed the Metabolic Capacity of the Intestinal Microbiota

The intestinal microbiota is involved in the metabolism, immune and neuromodulation of the organism and provides essential functions for its host (Adak and Khan, 2019). Stressors often affect their hosts by altering the function of the intestinal microbiota. Therefore, it is necessary to investigate the functional changes in the intestinal microbiota after *C. freundii* infection.

**Figure 2:**
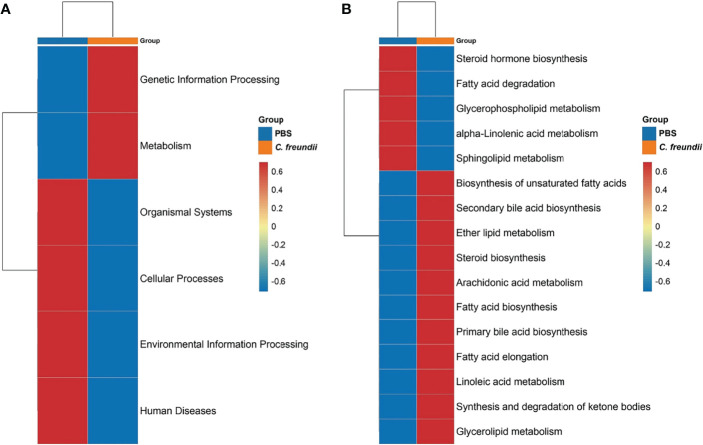
Prediction of intestinal microbiota function. **(A)** KEGG functional abundance statistics based on Tax4Fun. **(B)** Comparison of the abundance of lipid metabolism-related pathways. Representative values were normalized, colors indicate high (red) or low (blue) abundance of pathway annotations, and pathways were grouped by hierarchical clustering and illustrated at the right side of the heat map.

Tax4Fun was used to annotate the 16S rRNA gene sequence with KEGG function to obtain the annotation information of OTU at each KEGG functional level and the abundance of each function in different samples. The results showed that the abundance of metabolism and genetic information processing pathways increased after *C. freundii* infection, while the abundance of cellular processes, organismal systems, environmental information processing and human disease pathways decreased (**Figure 2A**). We further refined the comparison of metabolic pathways due to the highest abundance of metabolism-related pathways in both groups. The heat map showed that carbohydrate and amino acid-related metabolic pathways are not significantly altered (**Figure S2**). For lipid metabolism-related pathways, the abundance of steroid hormone biosynthesis, fatty acid degradation, glycerophospholipid metabolism, α-linolenic acid metabolism and sphingolipid metabolism pathways decreased after *C. freundii* infection, while the abundance of biosynthesis of unsaturated fatty acids, Fatty acid biosynthesis and other pathways increased (**Figure 2B**). Notably, the abundance of key metabolite bile acid synthesis-related pathways including primary bile acid biosynthesis and secondary bile acid biosynthesis increased after infection. The results of the functional annotation of the intestinal microbiota implied that *C. freundii* infection may affect the metabolism function of the intestinal microbiota and promote the synthesis of derived metabolites, especially bile acids.

### 3.3 Hepatopancreas RNA-Seq Quality Assessment and Determination of DEGs

Although *C. freundii* grows mainly in the crayfish intestine, it is usually isolated in the hepatopancreas, probably due to the increased permeability of the intestine which exposes the hepatopancreas to bacteria and their metabolites. Therefore, the hepatopancreas is also a potential organ of attack for *C. freundii*. To further investigate the potential effects of *C. freundii* infection on crayfish hepatopancreas, we constructed six hepatopancreas RNA libraries, and the control and infected groups contributed three of these libraries, respectively. The sequencing results for the six libraries were summarized in Table S2. An average of 45,205,366 raw reads were obtained, with Q20 and Q30 both above 93%. The final 43,371 unigenes with N50 of 2337 were generated by the Trinity assembly procedure. The length distributions of these assembled unigenes are summarized in **Figure S3A**. It is pleasing to note that the mapping rate of each sample ranged from 79.69% to 86.25%.

The assembled unigenes were presented for BLAST search against six public databases (Nr, Nt, Pfam, KOG/COG, Swiss-Prot, KEGG, and GO) to predict their possible functions. A total of 15,866 unigenes were annotated. A Venn diagram showed the count of annotations in some database (**Figure S3B**). The cellular process, membrane part and binding were the most annotated in the GO database under the terms BP, CC and MF, respectively. In the KEGG database, signal transduction received the highest number of annotations (1039) and the lowest number of biosynthesis of other secondary metabolites (14) (**Figures S3C, D**).

The results of PCA analysis showed that the samples of the control group and the infected group were clustered respectively, indicating that the samples of the same group were more similar and different from the other group, which validated the rationality of the experimental design (**Figure S4A**). A total of 803 DEGs had 414 up-regulated as well as 389 down-regulated (**Figure S4B**). Cluster analysis showed that DEGs had significantly different expression patterns among different groups (**Figure S4C**).

### 3.4 Infection Increased Lipid Degradation in the Hepatopancreas

**Figure 3:**
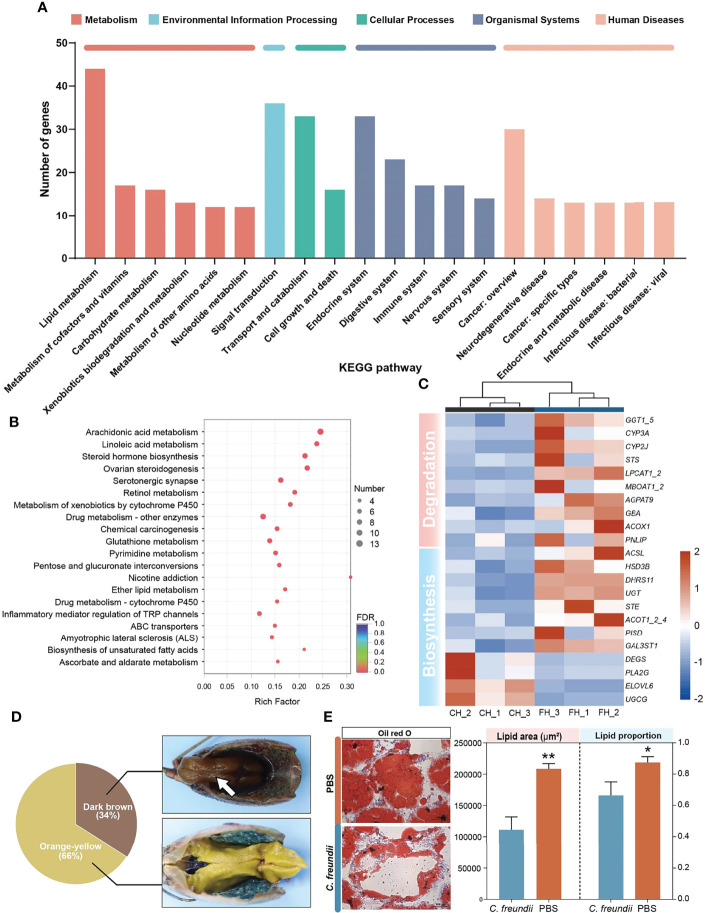
DEGs analysis and validation. **(A)** KEGG annotation of DEGs. **(B)** Top 20 pathways enriched in differentially expressed genes (DEGs) by KEGG. The color and size of the dots indicate FDR and DEG numbers, respectively. **(C)** Visual heat map of DEGs related to lipid metabolism based on expression data. **(D)** Gross observation of the hepatopancreas of crayfish. Dark brown hepatopancreas (White arrow). **(E)** Oil red O staining of the hepatopancreas in the two groups and lipid staining area and proportion of lipid to cell parenchyma (×200). *, **represent P < 0.05 and 0.01, respectively.

KEGG annotation results showed that lipid metabolism received the most differential gene annotations, followed by Signal transduction, Transport and catabolism and Endocrine system (**Figure 3A**). The distribution of the top 20 KEGG terms significantly enriched in the categories were shown in **Figure 3B**. Similar to the annotated results, KEGG enrichment analysis showed that differentially expressed genes in the hepatopancreas of crayfish infected with *C. freundii* were significantly enriched in lipid metabolism pathways, including Arachidonic acid metabolism, Linoleic acid metabolism, Steroid hormone biosynthesis, Ether lipid metabolism and Biosynthesis of unsaturated fatty acids. Considering the important role of the hepatopancreas in crayfish lipid metabolism, we hypothesized that *C. freundii* infection may have an effect on crayfish lipid metabolism. Further, differential genes mapping to lipid metabolism pathways were filtered out and an expression heat map was constructed. The results showed that the expression of all lipid degradation genes was significantly upregulated in the infected group, while the expression trend of biosynthesis-related genes was not uniform, implying that the lipid content in the hepatopancreas decreased after infection with *C. freundii* (**Figure 3C**). Gross observations also revealed that compared to the normal orange-colored hepatopancreas, a certain number of dark brown hepatopancreas appeared in the infected group (**Figure 3D**). To further support the conclusions obtained based on the transcriptome, lipids in the hepatopancreas were quantified by oil red O staining. The lipid droplet area and percentage of frozen hepatopancreatic tissue sections stained with oil red O showed a significant decrease trend with the invasion of *C. freundii* (**Figure 3E**). Transcriptome results suggest that *C. freundii* infection may lead to imbalance of lipid metabolism in crayfish hepatopancreas and induce lipid degradation.

### 3.5 Infection Caused Hepatopancreatic Cell Damage

**Figure 4:**
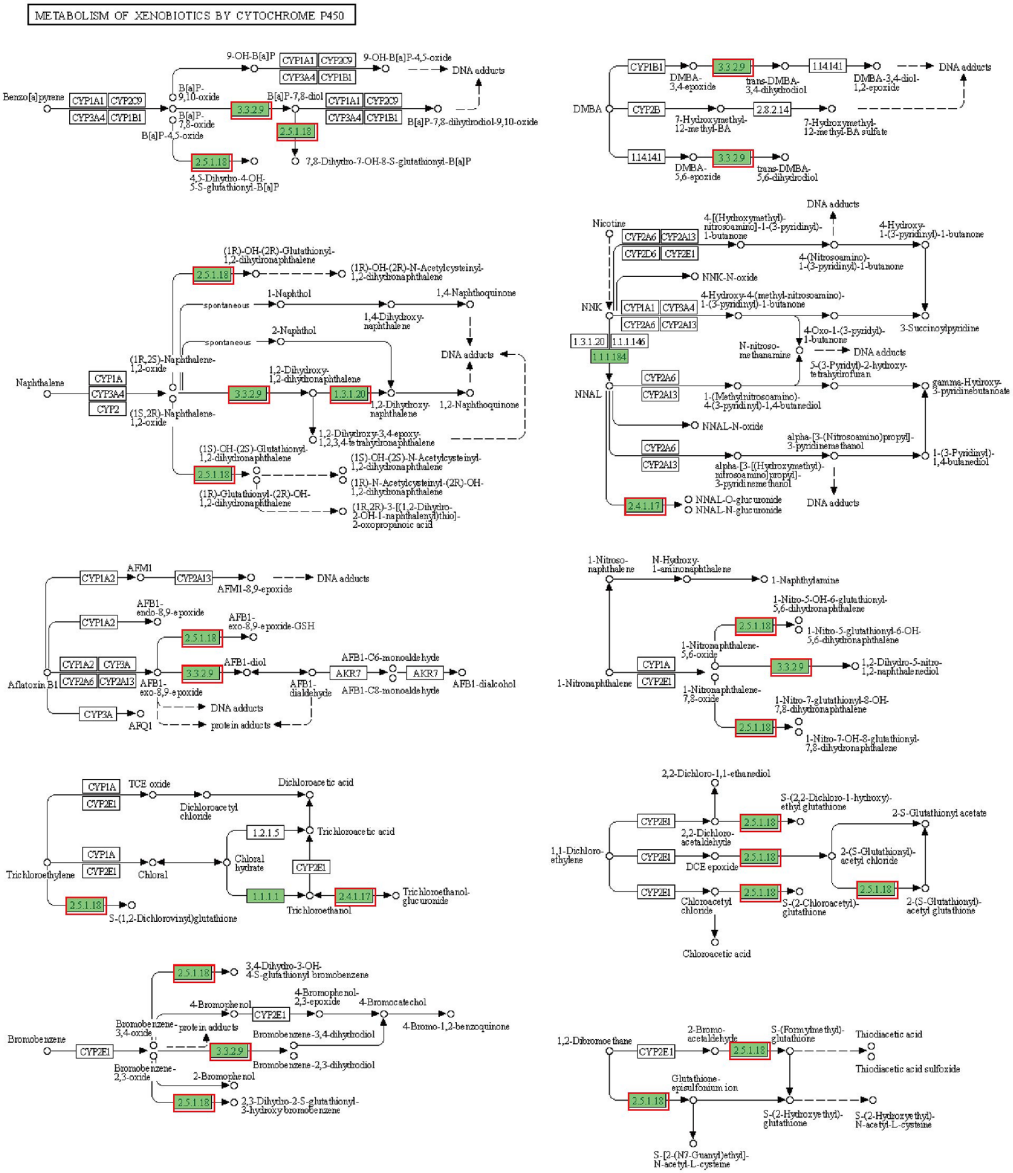
Metabolism of xenobiotics by cytochrome P450 pathway. The annotated enzyme is marked green. Enzymes in the red box are associated with up-regulated DEGs.

In addition to lipid metabolism, we also noted a significant enrichment of cytochrome P450-related pathways, including Metabolism of xenobiotics by cytochrome P450 and Drug metabolism - cytochrome P450 (**Figures 4** and **S5**). The annotated results suggest that the hepatopancreas may be exposed to bacterial metabolites of intestinal origin, as cytochrome P450 is involved in the metabolism of endogenous and exogenous substances. However, beyond its usual role in compound metabolism, the activity of cytochrome P450 can be deleterious since it can generate reactive oxygen species (ROS) (Cichoż-Lach and Michalak, 2014). Thus, all DEGs of cytochrome P450-related pathways were upregulated, suggesting that ROS may accumulate and lead to apoptosis in hepatopancreatic cells.

**Figure 5:**
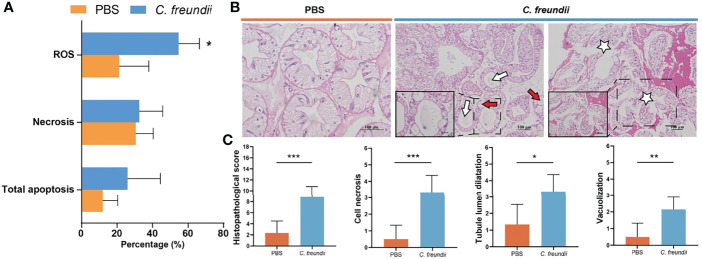
Analysis of damage to the hepatopancreas of crayfish. **(A)** ROS-positive, necrosis and apoptosis rates in hepatopancreatic cells. Results are shown as means ± SD. **(B)** Histopathological observations on the hepatopancreas of the crayfish (H&E staining). The lumen of hepatic tubules is enlarged and the wall of the tubules becomes thin (White arrow); Vacuolar degeneration of tube wall cells (Red arrow); Hepatopancreas cell necrosis (White Pentagram). **(C)** Intestinal histopathological score. (*:p < 0.05; **:p < 0.01; ***:p < 0.001).

To confirm our speculation, flow cytometry was used to evaluate apoptosis and ROS. According to the flow cytometry results, the percentage of apoptotic and necrotic cells was higher in the infected group. In addition, ROS in hepatopancreas were significantly higher in the infected group than in the control group, suggesting possible oxidative damage (**Figure 5A**).

The structural changes of hepatopancreas were evaluated by histology. The hepatopancreas from control group showed an intact tissue structure with a distinct star shape of the tubule lumen. In the infected group, a large amount of contents appeared in the hepatopancreatic lumen, the structure of the star-shaped lumen was disappeared, and the lumen of the hepatic tubules was enlarged and accompanied by thinning of the tubule wall. Additionally, cell necrosis, abscission and serious vacuolization of cells were also observed in infected hepatopancreas (**Figure 5B**). Histopathological scores showed that hepatopancreatic cell necrosis, tubule lumen dilatation and vacuolization were the most obvious and common pathological changes in infected crayfish, with highly significant differences in scores between the control and infected groups (**Figure 5C**). All of the above results indicated that the hepatopancreas was significantly damaged, which may be related to the activation of cytochrome P450-related pathway. The activation of the related pathway may be closely related to the entry of bacteria and their metabolites into the hepatopancreas due to dysbiosis of the intestinal microbiota.

## 4 Discussion

As a conditional pathogen that can infect aquatic organisms and mammals, *C. freundii* has become a threat to aquaculture activities and human health. In this study, crayfish was used as an infection host to provide new insights into the pathogenic mechanism of *C. freundii*. The results of the study showed that *C. freundii* disrupts the structure and function of the two main digestive organs of crayfish, the intestine and the hepatopancreas, through the intestine-liver axis.

As a prevalent Enterobacteriaceae bacterium in the natural environment, *C. freundii* is at risk of causing intestinal disease, as corroborated by the diarrheal symptoms that occur in humans after infection (Liu et al., 2018). Studies have shown that the intestinal microbiota responds to changes in the host’s own physiology and external stimuli (Piazzon et al., 2019). Pathogens, especially bacteria, are one of the important sources of stimulation. *Vibrio harveyi* infection has been reported to cause an increase in harmful genera and a decrease in beneficial genera in the intestinal microbiota of *Epinephelus coioides* (Xiao Joe et al., 2021). Similar to previous reports, in the present study, high-throughput sequencing revealed altered composition of the intestinal microbiota. Phenotypic predictions of intestinal microorganisms showed higher phenotypic abundance of potentially pathogenic and forms biofilms after *C. freundii* infection. The intestinal microbiota plays a fundamental role in the development of the host’s immune system, modulating T-cell repertoires and regulating the T helper (Th) cell profile (Cebra, 1999). In addition, cellular metabolites such as butyrate induce differentiation and amplification of colonic regulatory T cells (Atarashi et al., 2013). Therefore, intestinal microbiota disorders are often accompanied by intestinal inflammation. In addition to altered intestinal microbiota, we also observed inflammatory cell infiltration in the crayfish intestine after infection. Pathologic states, such as intestinal inflammation, are associated with a leaky epithelial barrier. An inflammatory microenvironment affects intestinal physical and chemical barriers by altering the structure and function of epithelial intercellular junctions through direct and indirect mechanisms (Luissint et al., 2016). Consistent with the speculation, it was observed by histology that the intestinal tissues of crayfish were structurally damaged after infection. Disruption of the intestinal barrier reduces the function of the intestine against commensal microbiota and invading pathogens and enhances pathogen susceptibility. The results of this study suggest that *C. freundii* infection causes disruption of the crayfish intestinal microbiota, which further promotes intestinal inflammation and intestinal structural disruption.

The homeostasis of the intestine-liver axis depends on the normal structure and function of the intestine, and disruption of the intestine leads to exposure of the hepatopancreas to intestinal bacteria and their metabolites. The composition of bile acids mediated by bacterial metabolism in the intestine and is intrinsically linked to host physiology and is receiving increasing attention as a potential mechanism for disease states. In the intestine, primary bile acids are converted into more hydrophobic secondary bile acids, such as lithocholic acid and deoxycholic acid (LCA and DCA), mainly by bile salt hydrolases (BSH) (Chiang, 2013). It has been shown that Bacteroidetes is the dominant phyla in the distribution of BSH (Jones et al., 2008). The present study also found an increase in the relative abundance of Bacteroidetes in the infected group. Bile acid homeostasis in organisms is primarily regulated by the farnesoid X receptor (FXR) and G protein-coupled bile acid receptor 1 (GPBAR1). In addition to maintaining bile acid homeostasis, the above two receptors are involved in anti-inflammatory and intestinal barrier construction (Inagaki et al., 2006; Pols et al., 2011). However, some secondary bile acids, such as deoxycholic acid, can inhibit FXR signaling, leading to increased bile acid synthesis (Jiao et al., 2018). Moreover, FXR expression is also negatively regulated by inflammation. In the present study, we found that the increased abundance of bacteria transforming bile acids at the Phylum level was associated with an upregulation of the annotation of related pathways. This implies that there exists the possibility of increased secondary bile acids exacerbating inflammation and intestinal destruction.

Bile acids promote the hydrolysis of fats by emulsifying fats and increasing the contact area with lipase to enhance the activity of metabolic enzymes. We observed a decrease in hepatopancreas lipids after infection by oil red O staining. The entry of bile acids into the hepatopancreas through the damaged intestine-liver axis is one of the plausible explanations. We also found a discoloration of the hepatopancreas of crayfish in the course of the experiment. Studies have shown that crustaceans have less lipids and more bile acids in their dark brown hepatopancreas (Zhang et al., 2022). Hepatopancreas transcriptome results showed significant enrichment of cytochrome P450-related pathways. Cytochrome P450 is involved in the metabolism of endogenous and exogenous substances, suggesting the possibility of intestinal microbiota metabolites entering the hepatopancreas. In addition, overexpression of cytochrome P450 leads to ROS production and further results in altered mitochondrial permeability and transition potential (Cichoż-Lach and Michalak, 2014). These changes induce the development of apoptosis. An increase in ROS and apoptosis in the hepatopancreas of the infected group was also found in this study. Damage to the hepatopancreas structure was observed by histology. Furthermore, in addition to the activated cytochrome P450-related pathway, bile acids can also directly damage the hepatopancreas. Experimentally, hydrophobic bile acids are known to induce injury to hepatocytes (Liu et al., 2015). Direct induction of mitochondrial reactive oxygen species (mROS) and subsequent mitochondrial oxidative stress to initiate apoptosis has also been shown to be one of the mechanisms by which hydrophobic bile acids contribute to liver disease (Li et al., 2017). Thus, in addition to the destruction of the intestinal microbiota, bile acids are involved in the pathogenic process of *C. freundii* by causing hepatopancreatic damage through the damaged intestine-liver axis.

In conclusion, this study shows that *C. freundii* infection disrupts the intestinal microbiota and promotes pathogenic bacterial colonization and bile acid synthesis, which cause intestinal inflammation and damage tissue structure. Pathogenic bacteria and metabolites enter the hepatopancreas through the intestine-liver axis and cause lipolysis and tissue damage. The results of this study elucidate the effects of *C. freundii* on the hepatopancreas and intestine of crayfish and provide a reference for studying the pathogenic mechanism of *C. freundii*.

## Data Availability Statement

The data presented in the study are deposited in the Sequence Read Archive (SRA) at the NCBI repository, accession number PRJNA755475 and PRJNA755431

## Ethics Statement

The animal study was reviewed and approved by Animal Care and Use Committee of Sichuan Agricultural University.

## Author Contributions

ML, XH, and LL conceived and designed the research. ML, JW and HD performed the research and acquired the data. DC, YO, YG and SY analyzed and interpreted the data. All authors were involved in drafting and revising the manuscript. All authors contributed to the article and approved the submitted version.

## Funding

This research was supported by Sichuan Science and Technology Program (2021JDRC0125), Fund of Sichuan key R & D program key technology project (2020YFN0060), Fund of Chengdu Science and Technology Bureau key Research and development support plan (NO.2019-YF05-02018-SN, 2022-YF05-00636-SN), Sichuan Science and technology plan project (NO.2022NZZJ0014).

## Conflict of Interest

The authors declare that the research was conducted in the absence of any commercial or financial relationships that could be construed as a potential conflict of interest.

## Publisher’s Note

All claims expressed in this article are solely those of the authors and do not necessarily represent those of their affiliated organizations, or those of the publisher, the editors and the reviewers. Any product that may be evaluated in this article, or claim that may be made by its manufacturer, is not guaranteed or endorsed by the publisher.
