# Supplementary material for: The Damage of the Crayfish (Procambarus Clarkii) Digestive Organs Caused by Citrobacter Freundii Is Associated With the Disturbance of Intestinal Microbiota and Disruption of Intestinal-Liver Axis Homeostasis
Source: Front Cell Infect Microbiol. 2022 Jul 5;12:940576. doi: 10.3389/fcimb.2022.940576 (PMC9295903; doi:10.3389/fcimb.2022.940576)
Supplement: Supplementary file 1 [file Image_1.pdf]

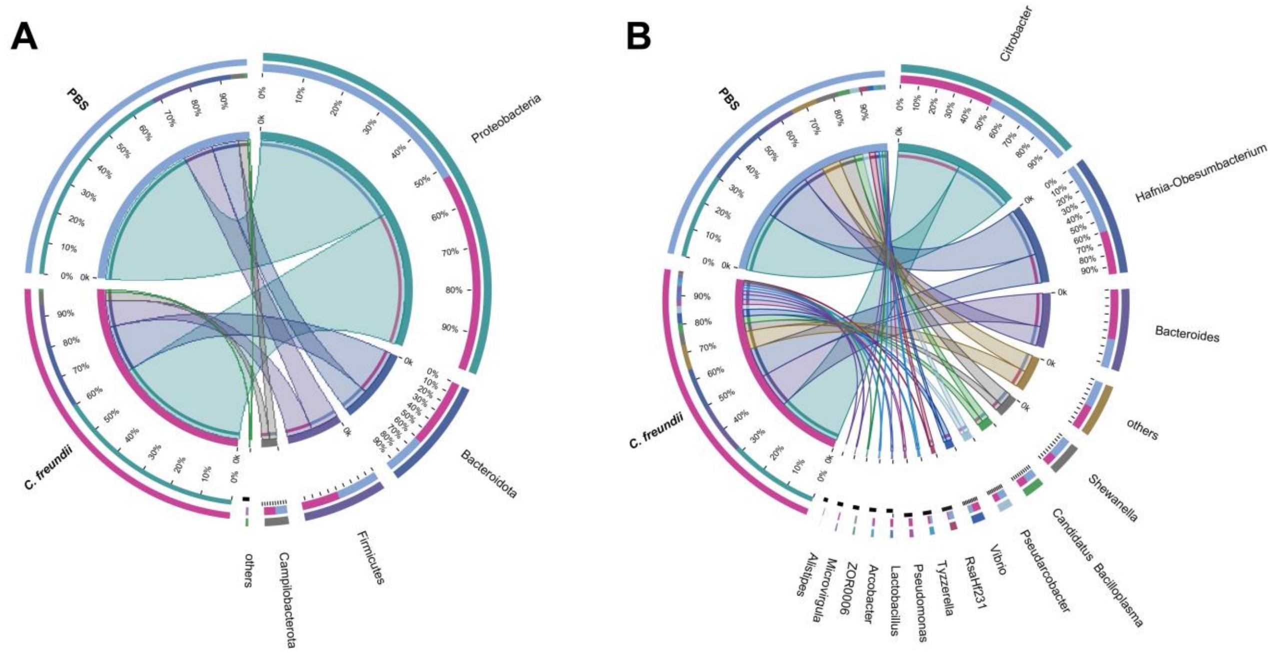

Figure S1. Composition of crayfish intestinal microbiota at the phylum and genus level. (A-B) Intestinal microbiota composition at the phylum and genus level in different groups of the crayfish, respectively.
