# Supplementary material for: The Damage of the Crayfish (Procambarus Clarkii) Digestive Organs Caused by Citrobacter Freundii Is Associated With the Disturbance of Intestinal Microbiota and Disruption of Intestinal-Liver Axis Homeostasis
Source: Front Cell Infect Microbiol. 2022 Jul 5;12:940576. doi: 10.3389/fcimb.2022.940576 (PMC9295903; doi:10.3389/fcimb.2022.940576)
Supplement: Supplementary file 2 [file Image_2.pdf]

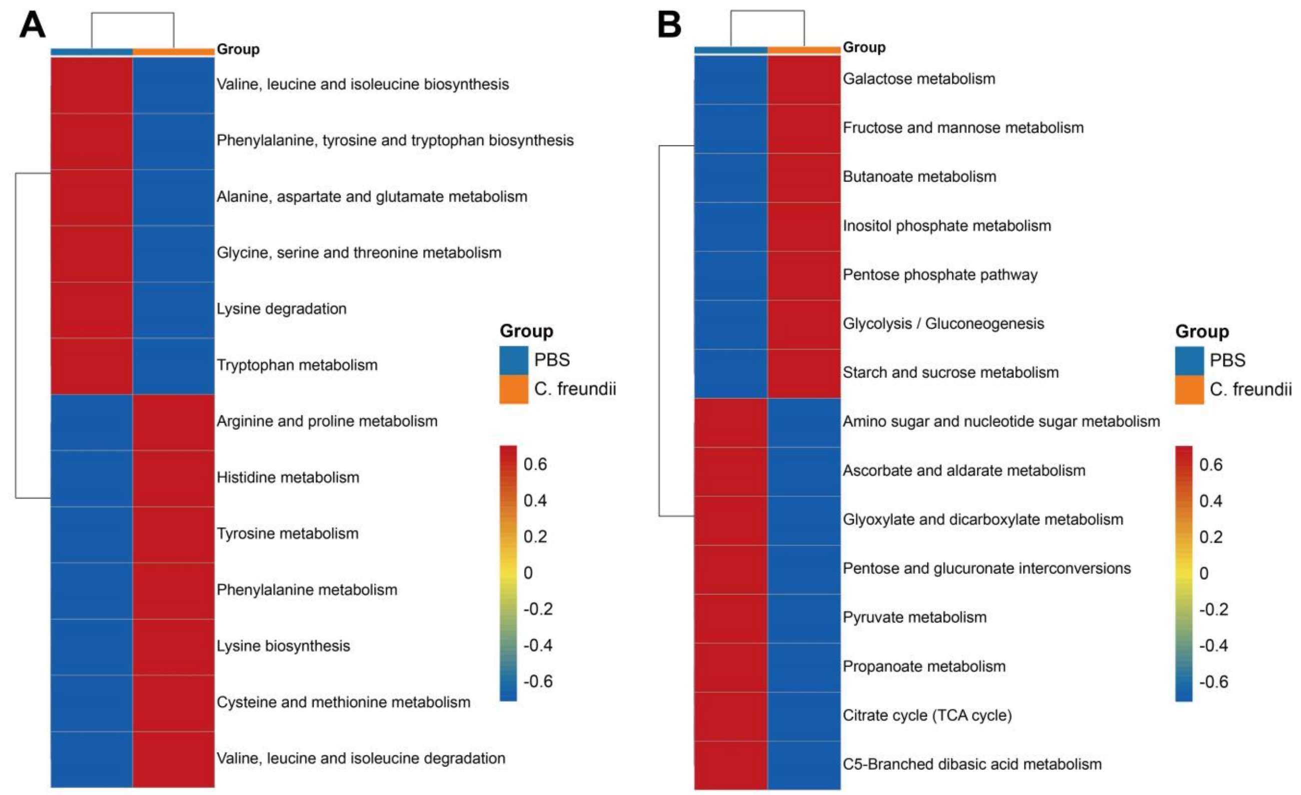

Figure S2. Comparison of amino acid (A) and carbohydrate (B) related metabolic pathways in the intestinal microbiota. Representative values were normalized, colors indicate high (red) or low (blue) abundance of pathway annotations, and pathways were grouped by hierarchical clustering and illustrated at the right side of the heat map.
