# Supplementary material for: The Damage of the Crayfish (Procambarus Clarkii) Digestive Organs Caused by Citrobacter Freundii Is Associated With the Disturbance of Intestinal Microbiota and Disruption of Intestinal-Liver Axis Homeostasis
Source: Front Cell Infect Microbiol. 2022 Jul 5;12:940576. doi: 10.3389/fcimb.2022.940576 (PMC9295903; doi:10.3389/fcimb.2022.940576)
Supplement: Supplementary file 3 [file Image_3.pdf]

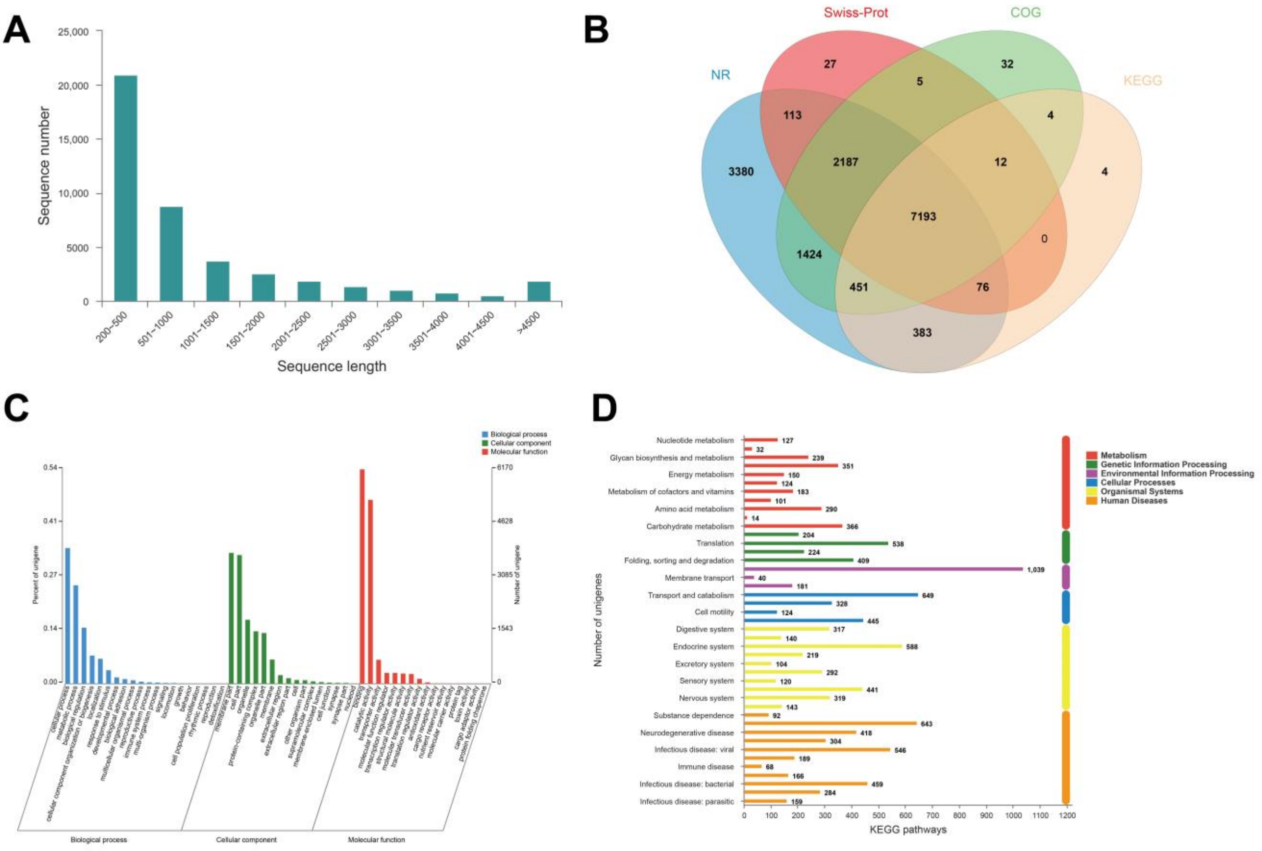

Figure S3. Characteristics of hepatopancreatic RNA-Seq. A: Length distribution of unigenes in the hepatopancreas transcriptomes. The X-axis represents the length of unigenes, and the Y-axis stands for the number of unigenes. **B**: Numbers of annotated unigenes in four public databases. **C**: Gene ontology (GO) annotations of the assembled unigenes. Each annotated sequence is assigned at least one GO term of the following: biological process, cellular component, or molecular function. **D**: Cluster of KEGG Ortholog database annotations of assembled unigenes.
