# Supplementary material for: The Damage of the Crayfish (Procambarus Clarkii) Digestive Organs Caused by Citrobacter Freundii Is Associated With the Disturbance of Intestinal Microbiota and Disruption of Intestinal-Liver Axis Homeostasis
Source: Front Cell Infect Microbiol. 2022 Jul 5;12:940576. doi: 10.3389/fcimb.2022.940576 (PMC9295903; doi:10.3389/fcimb.2022.940576)
Supplement: Supplementary file 4 [file Image_4.pdf]

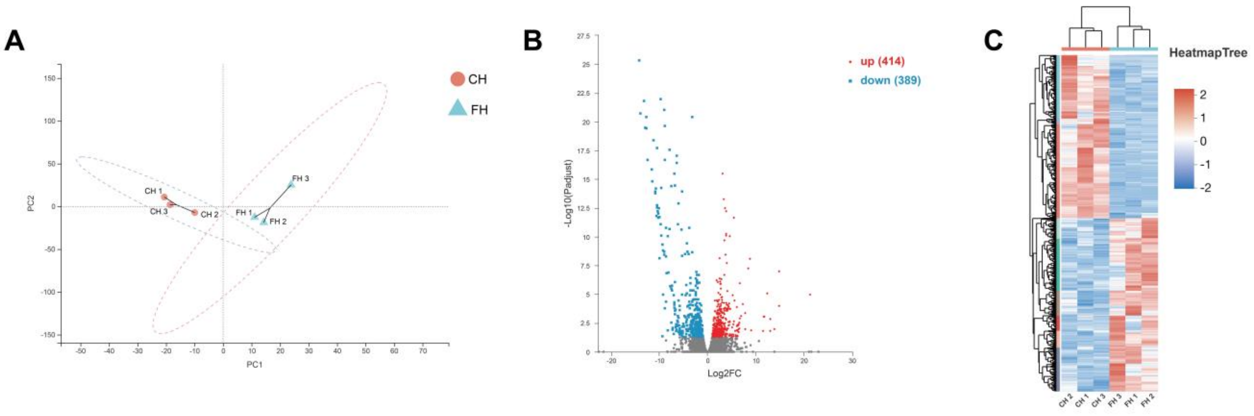

Figure S4. Differential analysis of transcriptome between control and infected groups. A: Principal component analysis (PCA) between control and infected samples. Blue triangles indicate samples infected with C. freundii, while red circles indicate samples without C. freundii infection. **B**: Volcano plot showing the distributions of the differentially expressed genes (DEGs). Each dot represents a gene. Red and blue dots represent up- and down regulated DEGs, respectively. **C**: Heatmap of differentially expressed gene expression patterns between control and infected samples.
