# Supplementary material for: The Damage of the Crayfish (Procambarus Clarkii) Digestive Organs Caused by Citrobacter Freundii Is Associated With the Disturbance of Intestinal Microbiota and Disruption of Intestinal-Liver Axis Homeostasis
Source: Front Cell Infect Microbiol. 2022 Jul 5;12:940576. doi: 10.3389/fcimb.2022.940576 (PMC9295903; doi:10.3389/fcimb.2022.940576)
Supplement: Supplementary file 5 [file Image_5.pdf]

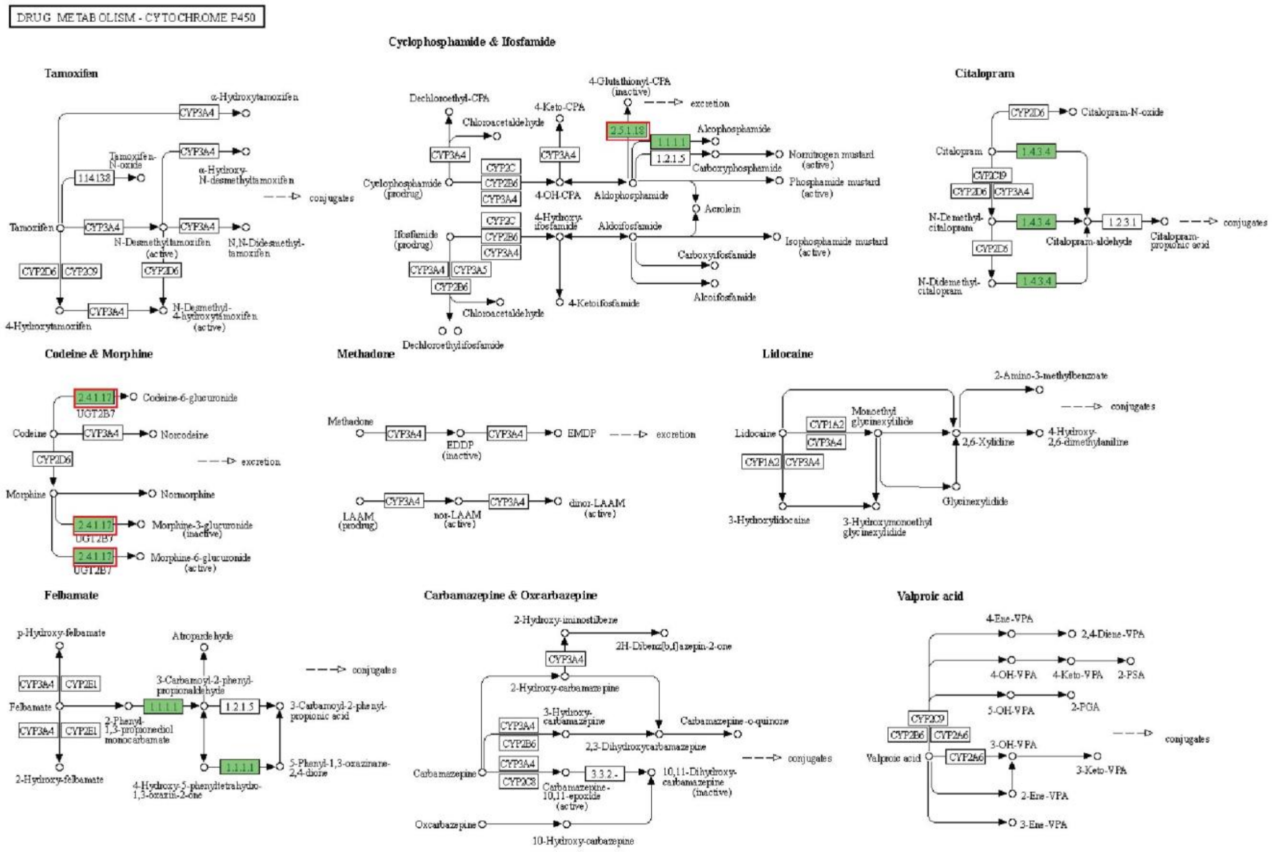

Figure S5. Drug metabolism - cytochrome P450 pathway. The annotated enzyme is marked green. Enzymes in the red box are associated with up-regulated DEGs.
