# Supplementary material for: The Damage of the Crayfish (Procambarus Clarkii) Digestive Organs Caused by Citrobacter Freundii Is Associated With the Disturbance of Intestinal Microbiota and Disruption of Intestinal-Liver Axis Homeostasis
Source: Front Cell Infect Microbiol. 2022 Jul 5;12:940576. doi: 10.3389/fcimb.2022.940576 (PMC9295903; doi:10.3389/fcimb.2022.940576)
Supplement: Supplementary file 6 [file Table_1.docx]

**Table S1.** Alpha diversity index of intestinal microbiota of crayfish injected with PBS and *C. freundii*.

| Samples | Community diversity | |  | Community richness | |
| --- | --- | --- | --- | --- | --- |
|  | Shannon | Simpson |  | Ace | Chao |
| PBS | 2.24±0.71 | 0.25±0.16 |  | 286.43±42.08 | 279.80±42.85 |
| *C. freundii* | 2.38±0.80 | 0.22±0.18 |  | 311.97±25.76 | 274.94±52.29 |
