# Supplementary material for: The Damage of the Crayfish (Procambarus Clarkii) Digestive Organs Caused by Citrobacter Freundii Is Associated With the Disturbance of Intestinal Microbiota and Disruption of Intestinal-Liver Axis Homeostasis
Source: Front Cell Infect Microbiol. 2022 Jul 5;12:940576. doi: 10.3389/fcimb.2022.940576 (PMC9295903; doi:10.3389/fcimb.2022.940576)
Supplement: Supplementary file 7 [file Table_2.docx]

**Table S2.** Raw and clean reads generated for the muscle transcriptomes

| **Sample** | **Raw reads** | **Clean reads** | **Clean Read Ratio** | **Q20 (%)^*^** | **Q30 (%)^**^** | **GC content (%)** |
| --- | --- | --- | --- | --- | --- | --- |
| CH_1 | 44738690 | 44460290 | 0.993777 | 98.11 | 94.15 | 47.45 |
| CH_2 | 48217116 | 47867316 | 0.992745 | 97.91 | 93.7 | 47.8 |
| CH_3 | 43994692 | 43664578 | 0.992497 | 97.86 | 93.6 | 47.48 |
| FH_1 | 47106282 | 46801002 | 0.993519 | 98.1 | 94.17 | 48.2 |
| FH_2 | 42830006 | 42562912 | 0.993764 | 98.12 | 94.21 | 47.96 |
| FH_3 | 44345410 | 43986678 | 0.991911 | 98.2 | 94.45 | 49.67 |

^*^Percentage of bases with quality score ≥ 20 (bases with accuracy of 99%).

^**^Percentage of bases with quality score ≥ 30 (bases with accuracy of 99.9%).
